# Comparison of the ankle-brachial index with parameters of stiffness and peripheral arterial resistance assessed by photoplethysmography in elderly patients

**DOI:** 10.1590/1677-5449.180084

**Published:** 2019-05-15

**Authors:** Kelser de Souza Kock, João Batista Ferreira da Silva, Jefferson Luiz Brum Marques

**Affiliations:** 1 Universidade do Sul de Santa Catarina – UNISUL, Tubarão, SC, Brasil.; 2 Universidade Federal de Santa Catarina – UFSC, Florianópolis, SC, Brasil.

**Keywords:** photoplethysmography, the elderly, hypertension, atherosclerotic, ankle-brachial index

## Abstract

**Background:**

The ankle-brachial index (ABI) uses the ratio between systolic blood pressures at the ankle and the arm to diagnose peripheral arterial disease (PAD) noninvasively. Photoplethysmography (PPG) measures and records changes to the blood volume in the human body using optical techniques.

**Objectives:**

The objective of this study was to compare ABI with arterial stiffness and peripheral resistance parameters assessed using PPG in elderly patients and to propose a model for prediction of ABI.

**Methods:**

A cross-sectional, quantitative study was conducted. The sample comprised elderly patients seen at a medical specialties clinic at the Universidade do Sul de Santa Catarina (UNISUL), Brazil. Age, sex, body mass index (BMI), comorbidities, smoking, and physical activity were recorded. The variables obtained using PPG and ABI were compared using bivariate and multivariate linear regression, with an α error of 0.05.

**Results:**

A total of 93 elderly patients were assessed, 63.4% of whom were women. In 98.9% of cases, ABI was within normal limits. Comparison of ABI with variables acquired by PPG revealed significant associations with age. However, no significant associations were observed between ABI and PPG. The multivariate model indicated that only age, sex, and smoking were associated with ABI.

**Conclusions:**

In conclusion, ABI and PPG exhibited associations with arterial aging, considering its correlation with age. However, ABI was only related to age, sex, and smoking. More studies are needed to evaluate the potential uses of PPG for screening for vascular diseases in ambulatory settings.

## INTRODUCTION

Biomedical technology seeks new equipment and new techniques that can offer more precise, early, and noninvasive diagnoses for assessment of peripheral arterial disease (PAD).^1^ Against this background, arterial health, which is related to the greatest cause of morbidity and mortality in the modern world, can be estimated using plethysmography, an examination employing the photoplethysmography (PPG) technique, which measures and records changes to the blood volume in the human body.^2^ This examination is promising in the global scenario, enabling risk stratification of cardiovascular diseases, nut it is as yet little used in clinical practice.^3^

The term plethysmography is a combination of two Greek words: “plethysmos”, which means increase, and “graph”, which means write, and it refers to an instrument primarily employed to determine and record variations in blood volume or blood flow in the body with each heartbeat.^3^ Photoplethysmography employs a light source and a sensor to analyze the cardiovascular pulse wave as it propagates through the body.^3^ The PPG signal reflects the circulation of the blood, which goes from the heart to the tips of the fingers and toes via the blood vessels in an undulating movement, and can be used to estimate arterial stiffness and cardiovascular aging and, potentially, detect atherosclerosis.^3^^,^ 4 It is an optical measurement technique that employs invisible infrared light emitted into the tissues, and the quantity of backscattered light corresponds to the variation in blood volume.^3^^,^ 5 ^,^ 6

However, capillary blood volume monitored by PPG must be evaluated by mathematical analysis, including differential and integral calculations, to compose the first and second derivatives and the area of the original pulse. This is why analysis is conducted using computational algorithms that are able to identify the characteristics of the pulse, such as systolic peak, dicrotic wave, and diastolic peak. The technique is also used to measure important characteristics of blood flow acceleration in a test known as acceleration plethysmography (APG).^3^

Considering these characteristics, PPG could be used as a method for early and noninvasive detection of vascular function abnormalities indicative of increased risk of atherosclerosis in certain population groups, such as the elderly.^6^ In this age group, which comprises people aged 60 years or over,^7^^,^ 8 it is common for morbidities to coexist and conditions such as diabetes mellitus, systemic arterial hypertension (SAH), arteriosclerosis, atherosclerosis (all related to arterial stiffness) are of particular concern.^9^

Thus, noninvasive assessment of subclinical arteriosclerosis in hypertense patients could provide a foundation for therapeutic management and contribute to prevention of secondary cardiovascular complications. Nowadays, there are several different methods that can be used to evaluate changes to the functional properties of the arteries, to determine the arterial stiffness index and, consequently, to detect subclinical atherosclerosis.^6^

One widely-used method that provides quantitative data for diagnosis of atherosclerotic disease is the ankle-brachial index (ABI).^10^ This indicator has a robust association with the severity of atherosclerosis in the carotid and coronary arteries and with PAD and thus with risk of mortality from cardiovascular and cerebrovascular problems. The incidence of vascular pathologies is increasing all over the world and is the result of many different factors, such as aging of the population, inactivity, smoking, alcoholism, stress, genetic factors, and hypercaloric nutrition.^10^^,^ 11

Operationally, ABI utilizes the ratio between systolic blood pressure (SBP) in the ankle and the arm, and is a simple, noninvasive and low cost method that is very reliable for diagnosing PAD.^10^^,^ 12 ^-^ 14 In order to calculate ABI, the highest SBP reading from the posterior tibial artery or the dorsal artery of the foot (measured in both limbs or just one, depending on causality) is divided by the highest systolic pressure reading from the brachial arteries.^1^^,^ 11 ^,^ 14

Many different studies conducted all over the world have demonstrated that ABI can be considered an effective noninvasive tool for diagnosis of carotid atherosclerotic disease, because it has the capacity to detect variations in flow generated by stenosis (moderate or severe). Normal ABI values are in the range of 0.91 to 1.30; results greater than 1.30 or lower than 0.91 are considered strongly predictive of diffuse atherosclerotic disease and demonstrate the presence of arterial stiffness caused by calcification of the tunica media and consequent rigidity of the vessel wall.^10^^,^ 15

Against this background, the objective of this study was to compare ABI of elderly patients with parameters of arterial stiffness and peripheral resistance assessed using PPG. A secondary objective was to propose a model for prediction of ABI in elderly people.

## METHODOLOGY

A cross-sectional, quantitative study was conducted with patients seen at the medical specialties clinic at the Universidade do Sul de Santa Catarina (UNISUL), Brazil.

People over the age of 60 years who agreed to take part in the study were enrolled from January 2017 to March 2018. Patients with psychomotor agitation that could interfere with the quality of the PPG signal were excluded from the study.

The sample size was estimated at n ≥ 85, based on an α error of 0.05, a β error of 0.20, and a correlation of r = 0.3, according to the following Expression (1) 16:

$$n\geq {\left\{(Z_{1-}{}_{a/2}+Z_{1-}{}_{b})/\left[1/2*\ln\left(1+r\right)/\left(1-r\right)\right]\right\}}^{2}+3$$ (1)

People who agreed to take part in the study were invited to attend for data collection at UNISUL and informed about the study before enrollment. They then signed free and informed consent forms. This project was approved by the UNISUL Research Ethics Committee, decision number 1.419.019, and granted approval certificate number 51217515.2.0000.5369.

At data collection, participants were requested to proceed to the study room and lie down in decubitus dorsal for 10 minutes to rest, when personal details were recorded before they were tested. Initially, age, sex, and body mass index (BMI), calculated as weight (kg)/height squared (m^2^), and presence of comorbidities, such as type 2 Diabetes mellitus (DM 2), SAH, dyslipidemia, heart diseases, and smoking, were recorded. In addition to these details, participants were asked whether or not they engaged in physical activity (PA) for at least 150 minutes per week.^17^^-^ 19 No symptoms of PAD were assessed.

Next, blood pressure (BP) was measured. An OMRON HEM 7113 automated oscillometric sphygmomanometer (AOS) was used.^20^^,^ 21 Measurements were taken at arms and ankles, starting with the right arm, followed by the left arm, right ankle, and left ankle. The ABI was calculated using the highest brachial SBP and the highest ankle SBP, according to the following Equation (2):^14^^,^ 18

$$ABI=\frac{Ankle\;SBP}{Brachial\;SBP}$$ (2)

Participants were then examined with a Reflex Aqwave pulse oximeter fitted to the index finger of the right hand, with capacity to store curve values at a sampling frequency of 60 Hz and a duration of 1 minute. Data were exported in a text file to MATLAB (Mathworks Inc., USA) software for analysis computational in.

The systolic peak and the diastolic peak of the pulse wave were identified in order to enable calculation of the stiffness index (SI) and the augmentation index (AI). After manipulation of the signal, the SI and AI were calculated as follows (3,4):

$$SI=\frac{height\left(m\right)}{\Delta T\left(s\right)}$$ (3)

$$AI=\frac{DP}{SP}*100\%$$ (4)

Where:

• height (m) is the height of the study participant;

• ΔT (s) is the time elapsed between systolic peak and diastolic peak;

• DP is the amplitude of the diastolic peak;

• SP is the amplitude of the systolic peak.

**Figure 1:**
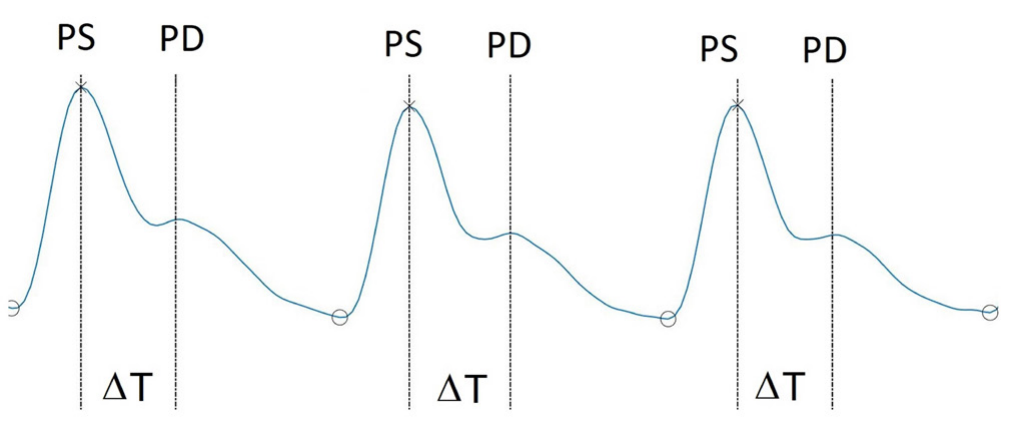
An example pulse curve obtained using PPG. SP = Systolic peak; DP = Diastolic peak; ΔT = Interval between systolic peak and diastolic peak.

Figure 1 illustrates the shape of a pulse wave and the variables ΔT, DP, and SP, needed to calculate SI and AI.

After information had been collected, data were tabulated and analyzed with Microsoft Excel and SPSS version 20.0. Quantitative variables were described using measures of central tendency and dispersion of data. Qualitative variables were described using absolute frequencies and percentages.

Comparisons of variables obtained with PPG (SI, ΔT, and AI) and ABI were performed using bivariate linear regression, considering an α error of 0.05. Multivariate linear regression was used with backward elimination for identification of variables associated with ABI, considering only those with p < 0.2. Residuals were analyzed with the Shapiro-Wilk test. Winsorization was used to exclude an ABI outlier to achieve a better fit.

## RESULTS

A total of 100 people were evaluated, 7 of whom were excluded because of poor quality PPG signals. Therefore, the sample comprised 93 people, with a median age of 66 years, the majority of whom were women. Median BMI was in the overweight range and SAH was the most prevalent comorbidity. Additional data are shown in Table 1.

**Table 1:** Epidemiological profile of the study participants.

	**Median (P25-P75)**
Age (years)	66.6 (62.8-75.1)
Sex#	
Male	34 (36.6%)
Female	59 (63.4%)
BMI (kg/m^2^)	27.0 (23.6-29.5)
HR (bpm)	65.4 (61.0-72.0)
brachial SBP (mmHg)	137.0 (124.5-153.0)
brachial DBP (mmHg)	76.0 (68.0-82.5)
SBP ankle (mmHg)	151.0 (138.5-164.0)
ankle DBP (mmHg)	75.0 (70.0-80.5)
Comorbidities#	
SAH	53.0 (57.0%)
DM	10 (10.8%)
Dyslipidemia	35 (37.6%)
Heart disease	22 (23.7%)
Smoking	11 (11.8%)
Physical activity	57 (61.3%)

# variable expressed as n (%);

BMI = Body mass index; HR = Heart rate; bpm = Beats per minute; SBP = Systolic blood pressure; DBP = Diastolic blood pressure; SAH = Systemic arterial hypertension; DM = Diabetes mellitus; P25: percentile 25; P75: percentile 75.

**Figure 2:**
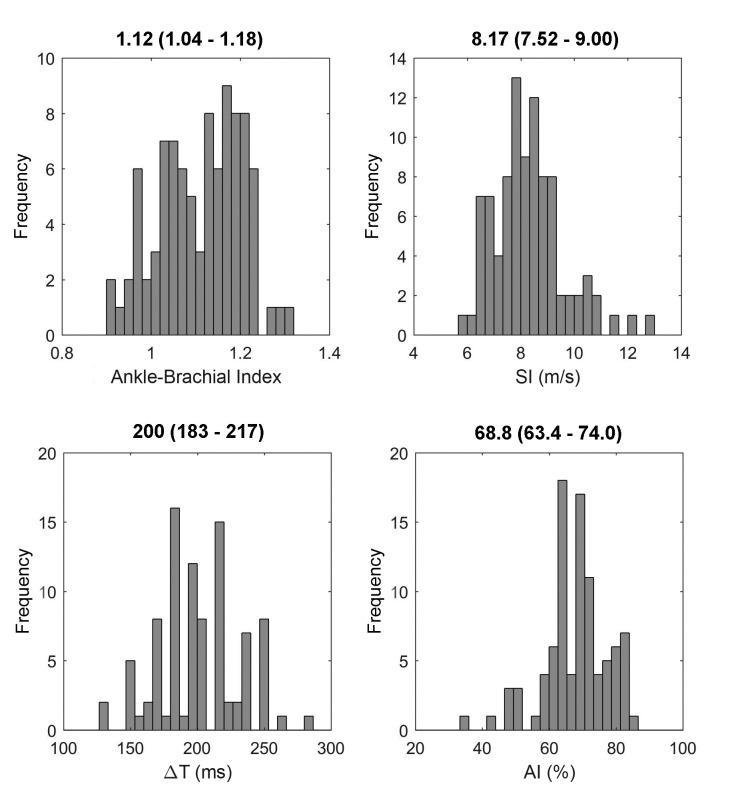
Graphs and medians (p25-p75) for ABI, SI, ΔT, and AI. SI = Stiffness Index; ΔT = Change in time; AI = Augmentation index.

With regard to ABI values, 92 cases (98.9%) were within the normal range, from 0.9 to 1.3. Just one case had an ABI of 0.56, and was excluded from the sample to standardize the results. Figure 2 shows graphs and medians (p25-p75) for ABI and for the variables obtained using PPG (SI, ΔT, and AI).

**Figure 3:**
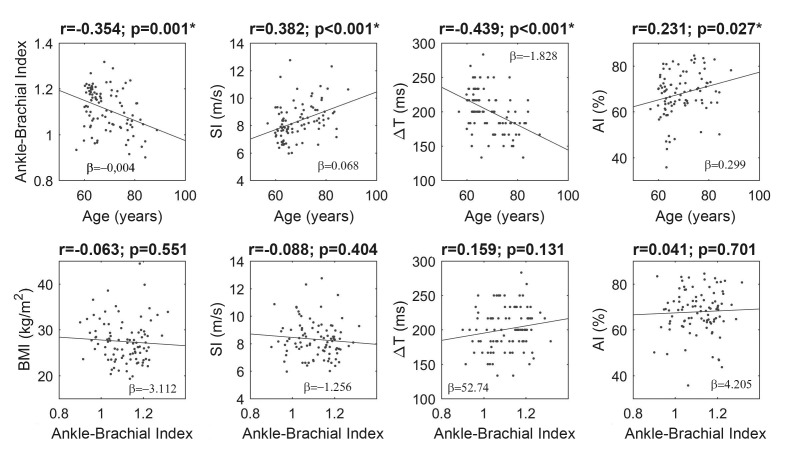
Correlations between ABI, SI, ΔT, AI, and BMI. BMI = Body mass index; SI = Stiffness index; ΔT = Change in time; AI = Augmentation index. *statistically significant values (p<0.05).

Comparison of variables obtained using PPG (SI, ΔT, and AI) and ABI in relation to age detected significant associations for all parameters, showing that the older the subject, the lower the ABI and ΔT and the higher the SI and AI. Comparisons of ABI with BMI and with variables extracted from PPG did not identify any significant correlations (Figure 3).

Preparatory to multivariate linear regression for analysis of variables potentially related to ABI, bivariate linear regression was conducted to define which variables would be used in the model. The results showed that only age, sex, and smoking were associated with the outcome ABI (Table 2). An r^2^ of 0.288 was obtained, with normal residuals (p = 0.296), and no collinearity.

**Table 2:** Bivariate and multiple linear regressions of variables related to the ABI of the study participants.

	**β (95%CI)**	**p**	**β (95%CI)**	**p**
Age (years)	-0.004 (-0.007;-0.002)	0.001	-0.005 (-0.007;-0.003)	<0.001
Sex#	-0.059 (-0.097;-0.021)	0.003	-0.060 (-0.094;-0.025)	0.001
BMI (kg/m^2^)	-0.001 (-0.006;0.003)	0.551		
SI (m/s)	-0.006 (-0.021;0.008)	0.404		
AI (%)	0.001 (-0.002;0.002)	0.701		
SAH*	-0.033 (-0.071;0.005)	0.088		
DM*	-0.032 (-0.094;0.029)	0.300		
Dyslipidemia*	0.007 (-0.032;0.047)	0.718		
Heart disease*	-0.016 (-0.061;0.029)	0.486		
Smoking*	-0.064 (-0124;-0.004)	0.038	-0.072 (-0.125;-0.018)	0.009
Physical activity*	0.024 (-0.015;0.063)	0.230		

# Male = 1; Female = 2;

* Absent = 0, Present = 1;

95%CI = 95% confidence interval; BMI = Body mass index; SAH = Systemic arterial hypertension; DM = Diabetes mellitus; AI = Augmentation index; SI = Stiffness index.

## DISCUSSION

The sample selected for this study predominantly comprised women, overweight people, and physically active people, and the principal comorbidity was SAH. In general, SAH is one of the most prevalent comorbidities among the elderly, and prevalence is even higher among women. The drop in steroidal hormone production after the menopause causes increased vascular tone of peripheral arteries, leading to SAH.^22^ The profile of this sample is in line with other studies, in which women seek health services more often than men, who very often only discover that they are hypertensive after a severe clinical event, such as a heart attack or stroke.^22^^,^ 23

Smoking is one of the most important risk factors of the principal causes of death among the elderly. The prevalence of smoking in the present study was very similar to the prevalence observed in the city of São Paulo, which was 12.2%.^24^ It is known that exposure to tobacco predisposes to occurrence of limiting and fatal diseases, such as peripheral vascular disease, cerebrovascular disease, heart diseases, and others. The elderly already have a higher prevalence of chronic conditions and when associated with smoking, the risk of members of this population developing chronic diseases increases.^23^

Physical inactivity is another factor related to occurrence of cardiovascular diseases.^25^ In the present study, more than half the sample were physically active, in contrast with global data showing that 70% of the population is sedentary. According to the World Health Organization (WHO), the ideal weekly level of exercise for adults is a minimum of 150 minutes of moderate PA or 75 minutes of intense PA.^26^ However, in the present study, PA level was not quantified objectively, which is a potential source of bias.

It is known that ABI is an important marker of PAD in both symptomatic and asymptomatic people and functions as a predictor of risk of cardiovascular diseases.^12^^,^ 17 In this study, the ABI value was within the normal range, in general. This is because of the profile of the sample studied, among whom there was a high proportion of people who are physically active, which is a protective factor, and many non-smokers, which is also a protective factor. The risk factors most associated with vascular disease are male sex, physical inactivity, smoking, advanced age, and SAH.^6^^,^ 17

In general, patients with a tendency to develop PAD can be sent for a noninvasive vascular examination so that they can be stratified more precisely, contributing to better diagnosis of disease.^27^ Such assessments can be achieved using tests based on physiological aspects, such as ABI, PPG, and pulse wave velocity (PWV), among others, and examinations of the anatomy, such as computed tomography angiography, magnetic resonance angiography, and duplex ultrasonography, which, in addition to anatomic information, also provide hemodynamic information.^27^

This study focused on tests that assess disease physiology, comparing ABI values with parameters obtained by PPG. The PPG parameters investigated were ΔT, SI, and AI. These indices do not have reference values, but are associated with arterial stiffness.^3^ Wowerm et al.^28^ conducted a study in which these and other PPG parameters were compared with AI and PWV obtained by applanation tonometry. The AI obtained by PPG was the variable most related to AI and PWV obtained by tonometry.

Pulse wave velocity can be assessed using the applanation tonometry method, in which pressure sensors are placed in superficial arteries to assess arterial stiffness, for example the carotid, radial, and femoral arteries (the carotid-femoral PWV is considered the gold standard).^28^ Although PWV is the gold standard for assessment of arterial stiffness, the tonometry method is inconvenient and difficult to operate. One alternative is PPG, which uses a less expensive technology and is not operator-dependent, making it feasible and appropriate for clinical practice.^28^^,^ 29

Pulse wave velocity is a biomarker of vascular degeneration. It monitors the cardiac heartbeat cycle, in which a pulse wave is generated and travels through the arterial bed until it meets peripheral resistance. Arterial resistance is dependent on the degree of a vessel’s complacency.^29^ Millasseau et al. has shown that PWV can be derived from parameters obtained by PPG.^30^ In younger people, vascular complacency is better because the arteries have greater elasticity, and so the reflected wave is slow, with a longer interval (ΔT) between systolic and diastolic pressure and a dicrotic notch.^29^ In older patients, vascular aging and comorbidities such as SAH and DM cause increased PWV, with a shorter ΔT and an less perceptible dicrotic notch.^29^ The SI is a parameter that normalizes ΔT according to a person’s height, with the same order (velocity) and units (m/s) as PWV.^3^

Thus, PWV is strongly correlated with age and BP, since both reduce vascular complacency, increasing arterial stiffness. Current SAH guidelines recommend using biomarkers to improve the accuracy of cardiovascular risk stratification.^29^

Comparison with age revealed that the older the patient, the worse the result obtained in relation to ABI reference values and also in relation to parameters obtained from PPG. This result shows that arterial stiffness index increases, demonstrating that these indicators could be used as markers of arterial aging.^2^

With regard to comparisons between ABI and PPG variables, no significant associations were observed. This is because of the profile of the patients studied, the majority of whom are in a risk group less prone to PAD, since median age was low and, as mentioned above, the older the age, the lower the ABI. Along the same lines, the percentage of smokers was small, and it is known that smoking also leads to reduced ABI.^2^ This shows that vascular disease has a direct relationship with age, in the sense that the older the person, the greater the tendency to develop atherosclerosis, which was what was observed with ABI and the PPG indices.^2^

In this study, it had been expected that the comparison between ABI and PPG parameters would reveal a strong correlation, which proved not to be possible because the sample comprised healthy individuals without severe peripheral disease and with ABI within normal limits. Although there was no significant correlation, there were associations between age, ABI, and PPG parameters, showing that PPG also provides evidence of arterial aging and indicating the further studies are needed to confirm these data.

In a manner similar to measuring ABI using AOS, PPG using a pulse oximeter could become an accessible instrument for clinical practice, since it is practical and simple to use, offering a noninvasive method for early detection of cardiovascular disease.^20^

Multivariate linear regression demonstrated that older women and smokers were more likely to have a lower ABI. These findings are confirmed in the literature, which shows that factors such as aging and a smoking habit are strongly associated with atherosclerosis. Additionally, in contrast with what was believed to be the case, women have PAD rates that are greater than or equal to those among men, primarily after the menopause, because of reduced hormone production.^1^^,^ 31 It had been expected that PPG parameters would be involved, but no association with ABI was observed. In contrast, a study by Allen et al.^2^ that also used techniques for extracting PPG signals, demonstrated good accuracy for stratification of patients with PAD.^21^

Limitations of this study include the non-prior evaluation of PAD symptoms and the normal ABI results of the majority of the sample, comprising healthy elderly people, which interfered with the comparison with PPG indices. Additionally, use of AOS to obtain BP data may have created a source of bias, although it is recommended and is simple to use.^20^

## CONCLUSIONS

It was demonstrated that the majority of ABI values were within normal limits. Although correlations were found between age, ABI, and PPG parameters, indicating arterial aging, ABI was not associated with PPG indicators. The predictive model showed that women, older people, and smokers had lower ABI.

Notwithstanding, the results of this study show that PPG has potential as a noninvasive technique. However, further studies are needed to standardize vascular assessment using the PPG curve so it can be used for diagnosis and screening of PAD.
