# Supplementary material for: Comparison of the ankle-brachial index with parameters of stiffness and peripheral arterial resistance assessed by photoplethysmography in elderly patients
Source: J Vasc Bras. 2019 May 15;18:e20180084. doi: 10.1590/1677-5449.180084 (PMC6634263; doi:10.1590/1677-5449.180084)
Supplement: Translation

# Translation: Comparação do índice tornozelo-braquial com parâmetros de rigidez e resistência arterial periférica avaliados por fotopletismografia em idosos

**Authors:** Kelser de Souza Kock, João Batista Ferreira da Silva, Jefferson Luiz Brum Marques

**Affiliations:** 1 Universidade do Sul de Santa Catarina – UNISUL, Tubarão, SC, Brasil.; 2 Universidade Federal de Santa Catarina – UFSC, Florianópolis, SC, Brasil.

**Keywords:** fotopletismografia, idoso, hipertensão, aterosclerótica, índice tornozelo-braço

## Abstract

**Contexto:**

O índice tornozelo-braquial (ITB) utiliza a razão entre a pressão arterial sistólica do tornozelo e do braço para diagnosticar de forma não invasiva a doença arterial periférica (DAP). A fotopletismografia (*photoplethysmography*, PPG) faz a medição e o registro das modificações de volume sanguíneo do corpo humano por meio de técnicas ópticas.

**Objetivos:**

O objetivo deste estudo foi comparar o ITB com parâmetros de rigidez arterial e resistência periférica avaliados pela PPG em idosos e propor um modelo de predição para o ITB.

**Métodos:**

Foi realizado um estudo transversal quantitativo. A amostra foi composta por idosos atendidos no ambulatório médico de especialidades da Universidade do Sul de Santa Catarina (UNISUL). Foram verificados: idade, sexo, índice de massa corporal (IMC), presença de comorbidades, tabagismo e atividade física. Para comparação das variáveis obtidas com a PPG com o ITB, foi realizada regressão linear bivariada e multivariada, considerando erro α = 0,05.

**Resultados:**

Foram avaliados 93 idosos, sendo 63,4% mulheres. Em 98,9% dos casos, o ITB apresentou-se dentro da normalidade. Na comparação do ITB e variáveis derivadas da PPG em relação à idade, foram demonstradas associações significativas. Contudo, não foram observadas associações significativas entre ITB e PPG. O modelo multivariado indicou que apenas idade, sexo e tabagismo foram associados ao ITB.

**Conclusões:**

Como conclusão, o ITB e a PPG demonstraram associação com o envelhecimento arterial, tendo em vista sua correlação com a idade; contudo, o ITB foi relacionado apenas com idade, sexo e tabagismo. Mais estudos são necessários para avaliar o potencial uso da PPG como rastreio de doenças vasculares em rotinas ambulatórias.

## INTRODUÇÃO

A tecnologia biomédica busca novos equipamentos e novas técnicas que permitam um diagnóstico mais preciso, precoce e não invasivo para avaliação da doença arterial periférica (DAP)^1^. Diante desse panorama, destaca-se a avaliação da saúde arterial, relacionada à principal causa de morbimortalidade do mundo moderno, que pode ser estimada através da pletismografia, exame que usa como técnica a fotopletismografia (*photoplethysmography,* PPG), a qual faz a medição e registro das modificações de volume sanguíneo do corpo humano^2^. Esse exame mostra-se promissor no cenário mundial, possibilitando a estratificação de risco das doenças cardiovasculares; contudo, ainda é pouco utilizado na prática clínica^3^.

A palavra pletismografia é uma combinação de duas palavras gregas: “plethysmos”, que significa aumento, e “graph”, que significa escrita, e é um instrumento usado principalmente para determinar e registrar as variações de volume de sangue ou o fluxo de sangue no corpo que ocorrem com cada batimento cardíaco^3^. A PPG usa uma fonte de luz e um detector para analisar a onda de pulso cardiovascular que se propaga através do corpo^3^. O sinal da PPG reflete a circulação do sangue, que vai do coração para as pontas dos dedos das mãos e pés através dos vasos sanguíneos em um movimento ondulatório, que pode ser usado para estimar a rigidez arterial e o envelhecimento cardiovascular e, potencialmente, detectar a aterosclerose^3^^,^ 4. É uma técnica de medida óptica que utiliza uma luz infravermelha invisível enviada para o tecido, sendo que a quantidade da luz retrodispersa corresponde à variação do volume de sangue^3^^,^ 5 ^,^ 6.

No entanto, a avaliação do volume de sangue capilar monitorada pela PPG exige análise matemática, incluindo cálculo diferencial e integral, para composição da primeira e da segunda derivadas e da área do pulso original. Por esse motivo, a análise é realizada por meio de algoritmos computacionais que conseguem identificar as características do pulso, como pico sistólico, onda dicrótica e pico diastólico. Além disso, são mensuradas características importantes da aceleração do fluxo sanguíneo, através de um exame denominado *acceleration plethysmography* (APG)^3^.

Nessa perspectiva, a PPG pode ser um método de detecção precoce e não invasivo das alterações funcionais vasculares indicativas de maior risco de aterosclerose em determinados grupos populacionais, como o dos idosos^6^. Nesse grupo etário, com idade igual ou superior a 60 anos^7^^,^ 8, é comum a sobreposição de morbidades, dentre as quais se destacam diabetes melito, hipertensão arterial sistêmica (HAS), arteriosclerose e aterosclerose, todas relacionadas com rigidez arterial^9^.

A avaliação não invasiva da arteriosclerose subclínica em pacientes hipertensos poderia, portanto, fundamentar bases terapêuticas e prevenir complicações cardiovasculares secundárias. Atualmente existem diferentes métodos que possibilitam avaliar alterações das propriedades funcionais das artérias, determinando-se o índice de rigidez arterial e, consequentemente, a aterosclerose subclínica^6^.

Um método muito utilizado que estabelece dados quantitativos para diagnóstico da doença aterosclerótica é o índice tornozelo-braquial (ITB)^10^. Esse indicador possui forte associação com a gravidade da aterosclerose nas artérias carótidas e coronárias e com DAP e risco de mortalidade nos problemas cardiovasculares e cerebrovasculares. O aumento de incidência de patologias vasculares ocorre em nível mundial, e deve-se a vários fatores, tais como envelhecimento da população, sedentarismo, tabagismo, etilismo, estresse, fatores genéticos e alimentação hipercalórica^10^^,^ 11.

Operacionalmente, o ITB utiliza a razão entre a pressão arterial sistólica (PAS) do tornozelo e do braço, sendo um método simples, não invasivo, de baixo custo e de grande confiabilidade para diagnosticar DAP^10^^,^ 12 ^-^ 14. Para o cálculo do ITB, é utilizada a relação da maior PAS da artéria tibial posterior e da artéria dorsal do pé (com obtenção nos dois membros ou em apenas um, dependendo da casualidade) com a maior pressão sistólica das artérias braquiais^1^^,^ 11 ^,^ 14.

Vários estudos têm demonstrado em todo o mundo que o ITB é considerado uma ferramenta não invasiva eficaz no diagnóstico de doença aterosclerótica carotídea, devido à sua capacidade em detectar variações de fluxo geradas por estenoses (moderadas ou graves). Valores de ITB entre 0,91 a 1,30 são considerados normais; os resultados acima de 1,30 ou abaixo de 0,91 constituem-se em fortes preditores de doença aterosclerótica difusa e demonstram a presença de enrijecimento arterial devido à calcificação da camada média e consequente rigidez da parede vascular^10^^,^ 15.

Nesse contexto, o objetivo desta pesquisa foi comparar o ITB de idosos com parâmetros de rigidez arterial e resistência periférica avaliados pela PPG. Além disso, buscou-se propor um modelo de predição para o ITB em idosos.

## METODOLOGIA

Foi realizado um estudo transversal, quantitativo com pacientes atendidos no ambulatório médico de especialidades da Universidade do Sul de Santa Catarina (UNISUL).

Foram incluídos indivíduos com idade superior a 60 anos que aceitaram participar do estudo no período de janeiro de 2017 a março de 2018. Foram excluídos do estudo pacientes com agitação psicomotora que pudesse influenciar a qualidade do sinal da PPG.

O tamanho da amostra foi estimado em n ≥ 85, baseando-se em um erro α = 0,05, erro β = 0,20 e nível de correlação r = 0,3, de acordo com a Expressão (1) 16:

$$n\geq {\left\{(Z_{1-}{}_{a/2}+Z_{1-}{}_{b})/\left[1/2*ln\left(1+r\right)/\left(1-r\right)\right]\right\}}^{2}+3$$ (1)

Os participantes que aceitaram participar do estudo foram convocados para a coleta de dados na UNISUL e informados do estudo antes da inclusão. Nesse momento foi realizada a assinatura do Termo de Consentimento Livre e esclarecido (TCLE). Este trabalho foi aprovado pelo Comitê de Ética em Pesquisa da UNISUL, sob o parecer 1.419.019, CAAE 51217515.2.0000.5369.

Na coleta de dados, os participantes eram orientados a irem até a sala de estudos e permanecerem deitados em decúbito dorsal por 10 minutos para um prévio descanso, enquanto seriam coletadas as informações pessoais e logo em seguida fossem submetidos aos testes. Inicialmente foram verificados idade, sexo, índice de massa corporal (IMC), calculado pela fórmula peso (kg)/altura (m^2^), e presença de comorbidades como diabetes melito tipo 2 (DM 2), HAS, dislipidemias, cardiopatias, tabagismo. Além disso, os participantes foram questionados se realizavam ou não atividade física (AF) durante pelo menos 150 minutos por semana^17^^-^ 19. Não foram avaliados sintomas de doença arterial periférica (DAP).

Após, foi realizada a mensuração da pressão arterial (PA). Foi utilizado um esfigmomanômetro oscilométrico automático (EOA) da marca OMRON HEM 7113^20^^,^ 21. As medidas foram realizadas nos braços e nos tornozelos, iniciando-se a aferição pelo braço direito, seguido pelo braço esquerdo, depois tornozelo direito e por fim tornozelo esquerdo. Para obtenção do ITB, foi considerada a maior PAS braquial e a maior PAS do tornozelo, de acordo com a seguinte Equação (2) 14 ^,^ 18:

$$ITB=\frac{PAS_{tornozelo}}{PAS_{braquial}}$$ (2)

Em seguida, os participantes foram examinados utilizando oxímetro de pulso da marca Reflex Aqwave no dedo indicador da mão direita, com capacidade de armazenamento dos valores da curva numa frequência amostral de 60 Hz e duração de 1 minuto. Os dados foram exportados em arquivo de texto para análise computacional no software MATLAB (Mathworks Inc., USA).

Para obtenção do índice de rigidez (IR) e do índice de aumento (IA), foram identificados o pico sistólico e o pico diastólico da onda de pulso. Após a manipulação do sinal, o IR e o IA foram calculados da seguinte forma (3,4):

$$IR=\frac{altura\left(m\right)}{\Delta T\left(s\right)}$$ (3)

$$IA=\frac{PD}{PS}*100\%$$ (4)

Onde:

• altura (m) é a altura do indivíduo participante do estudo;

• ΔT (s) é o tempo entre o pico sistólico e o pico diastólico;

• PD é a amplitude do pico diastólico;

• PS é a amplitude do pico sistólico.

A Figura 1 demonstra o formato de uma curva de pulso e a obtenção das variáveis ΔT, IR e IA.

**Figura 1:** Demonstração de uma curva de pulso obtida na PPG. PS = Pico sistólico; PD = Pico sistólico; ΔT = Intervalo entre pico sistólico e pico diastólico.

Após a coleta as informações, os dados foram tabulados e analisados com o Microsoft Excel e o programa estatístico SPSS versão 20.0. As variáveis quantitativas foram descritas por meio de medidas de tendência central e dispersão dos dados. As variáveis qualitativas foram descritas por meio de frequência absoluta e percentual.

Para comparação das variáveis obtidas com a PPG (IR, ΔT e IA) e o ITB, foi realizada regressão linear bivariada, considerando erro α = 0,05. Foi realizada regressão linear múltipla, utilizando o método *backward*, para identificação das variáveis associadas ao ITB, considerando apenas aquelas com p < 0,2. Os resíduos foram analisados pelo teste de Shapiro-Wilk. Foi realizada a *winsorização* para exclusão de um dado *outlier* do ITB visando a um melhor ajuste dos resultados.

## RESULTADOS

Foram avaliados 100 indivíduos, dos quais 7 foram excluídos devido à má qualidade do sinal da PPG. Assim, totalizou-se uma amostra de 93 sujeitos, com mediana de idade de 66 anos, na maior parte mulheres. A mediana do IMC encontrava-se na faixa do sobrepeso, e a HAS era a comorbidade mais prevalente. Outras informações podem ser visualizadas na Tabela 1.

**Tabela 1:** Perfil epidemiológico dos participantes do estudo.

	Mediana (P25-P75)
Idade (anos)	66,6 (62,8-75,1)
Sexo#	
Masculino	34 (36,6%)
Feminino	59 (63,4%)
IMC (kg/m^2^)	27,0 (23,6-29,5)
FC (bpm)	65,4 (61,0-72,0)
PAS braquial (mmHg)	137,0 (124,5-153,0)
PAD braquial (mmHg)	76,0 (68,0-82,5)
PAS tornozelo (mmHg)	151,0 (138,5-164,0)
PAD tornozelo (mmHg)	75,0 (70,0-80,5)
Comorbidades#	
HAS	53,0 (57,0%)
DM	10 (10,8%)
Dislipidemia	35 (37,6%)
Cardiopatia	22 (23,7%)
Tabagismo	11 (11,8%)
Atividade Física	57 (61,3%)

# variável descrita em n (%);

IMC = Índice de Massa Corporal; FC = Frequência Cardíaca; bpm = batimentos por minuto; PAS = Pressão Arterial Sistólica; PAD = Pressão Arterial Diastólica; HAS = Hipertensão Arterial Sistêmica; DM = Diabetes Melito; P25: percentil 25; P75: percentil 75.

No que se refere aos valores do ITB, 92 casos (98,9%) estavam dentro da faixa de normalidade, entre 0,9 e 1,3. Apenas um caso apresentou ITB de 0,56, e foi excluído da amostra para padronização dos resultados. A Figura 2 apresenta os histogramas e a mediana (p25-p75) do ITB e das variáveis obtidas com a PPG (IR, ΔT e IA).

**Figura 2:** Histograma e mediana (p25-p75) do ITB, IR, ΔT e IA. IR = Índice de Rigidez; ΔT = Variação de Tempo; IA = Índice de Aumento.

Na comparação das variáveis obtidas com a PPG (IR, ΔT e IA) e do ITB em relação à idade, foram demonstradas associações significativas em todos os parâmetros, demonstrando que, quanto maior a idade, menores o ITB e o ΔT e maiores o IR e o IA. Em se tratando da comparação do ITB com o IMC e as variáveis extraídas da PPG, não foi identificada correlação significativa (Figura 3).

**Figura 3:** Correlação entre ITB, IR, ΔT, IA e IMC. IMC = Índice de Massa Corpórea; IR = Índice de Rigidez; ΔT = Variação de Tempo; IA = Índice de Aumento. *valores estatisticamente significativos (p<0,05).

Na regressão linear múltipla para análise das variáveis relacionadas ao ITB, foi inicialmente realizada a regressão linear bivariada para estipular as variáveis pertinentes ao modelo. Ao final, apenas as variáveis idade, sexo e tabagismo mostraram-se associadas ao desfecho ITB (Tabela 2). Foi obtido um r^2^ = 0,288, com normalidade dos resíduos (p = 0,296) e ausência de colinearidade.

**Tabela 2:** Regressão linear bivariada e múltipla das variáveis relacionadas ao ITB dos participantes do estudo.

	β (IC95%)	p	β (IC95%)	p
Idade (anos)	-0,004 (-0,007;-0,002)	0,001	-0,005 (-0,007;-0,003)	<0,001
Sexo#	-0,059 (-0,097;-0,021)	0,003	-0,060 (-0,094;-0,025)	0,001
IMC (kg/m^2^)	-0,001 (-0,006;0,003)	0,551		
IR (m/s)	-0,006 (-0,021;0,008)	0,404		
IA (%)	0,001 (-0,002;0,002)	0,701		
HAS^*^	-0,033 (-0,071;0,005)	0,088		
DM^*^	-0,032 (-0,094;0,029)	0,300		
Dislipidemia^*^	0,007 (-0,032;0,047)	0,718		
Cardiopatia^*^	-0,016 (-0,061;0,029)	0,486		
Tabagismo^*^	-0,064 (-0124;-0,004)	0,038	-0,072 (-0,125;-0,018)	0,009
Atividade Física^*^	0,024 (-0,015;0,063)	0,230		

# Homem = 1; Mulher = 2;

* Ausência = 0; presença = 1;

IC95% = Intervalo de Confiança de 95%; IMC = Índice de Massa Corporal; HAS = Hipertensão Arterial Sistêmica; DM = Diabetes Melito; IA = Índice de Aumento; IR = Índice de Rigidez.

## DISCUSSÃO

A amostra obtida no presente estudo foi em sua maioria composta por mulheres, indivíduos com sobrepeso e praticantes de AF, e teve como principal comorbidade a HAS. De forma geral, a HAS é uma das comorbidades mais prevalentes em idosos, tendo prevalência ainda maior nas mulheres. Devido à queda na produção de hormônios esteroides pós-menopausa, ocorre o aumento do tônus vascular das artérias periféricas, provocando HAS^22^. O perfil da amostra corrobora outros estudos, nos quais as mulheres procuram mais os serviços de saúde se comparadas aos homens, os quais muitas vezes descobrem que são hipertensos somente após sofrerem um evento clínico grave, como infarto ou acidente vascular encefálico^22^^,^ 23.

O tabagismo é um dos fatores de risco mais importantes para as principais causas de morte entre os idosos. No presente estudo, a prevalência de tabagismo foi muito similar à prevalência na cidade de São Paulo, que foi de 12,2%^24^. Sabe-se que a exposição ao tabaco predispõe à ocorrência de doenças limitantes e fatais, como doenças vasculares periféricas, cerebrovasculares, cardiopatias, entre outras. Idosos já apresentam uma maior prevalência de agravos crônicos o que, quando associado ao tabagismo, aumenta o risco para essa população desenvolver doenças crônicas^24^.

A inatividade física também é um fator relacionado à ocorrência de doenças cardiovasculares^25^. No presente estudo, mais da metade da amostra era praticante de AF, diferente de dados mundiais demonstrando que 70% da população é sedentária. Segundo a Organização Mundial da Saúde (OMS) a prática ideal de exercícios semanal para adultos é de no mínimo 150 minutos de AF moderada ou de 75 minutos de AF intensa^26^. Contudo, na presente pesquisa, o nível de AF não foi quantificado de forma objetiva, sendo potencialmente um ponto de viés.

Sabe-se que o ITB é um importante marcador de DAP tanto em indivíduos sintomáticos quanto assintomáticos, servindo como preditor de risco para doenças cardiovasculares^12^^,^ 17. Neste estudo, o valor do ITB ficou dentro da faixa de normalidade, de forma geral. Isso se deve ao perfil dos indivíduos estudados, em que foi identificado um número mais expressivo de pacientes que praticavam AF, sendo este um fator protetor, e poucos tabagistas, o que também é um fator protetor. Por outro lado, os fatores de risco mais associados com doença vascular são sexo masculino, sedentarismo, tabagismo, idade avançada e HAS^6^^,^ 17.

De forma geral, os pacientes com uma tendência a desenvolver DAP podem ser submetidos a exame vascular não invasivo para que se possa estratificá-los de forma mais precisa, contribuindo para um melhor diagnóstico da doença^27^. Essas avaliações podem ser realizadas por testes que são baseados em aspectos fisiológicos, como o ITB, a PPG, a velocidade de onda de pulso (VOP), entre outros, e exames que avaliam a sua anatomia, como a angiografia por tomografia computadorizada, a angiografia por ressonância magnética e a ultrassonografia duplex, que fornece, além de informações anatômicas, também informações hemodinâmicas^27^.

Este estudo foi direcionado para exames que avaliam a fisiologia da doença, comparando valores de ITB com parâmetros derivados da PPG. Dentre os parâmetros da PPG avaliados, estão ΔT, IR e IA. Esses índices ainda não possuem valores de referência, mas estão associados com rigidez arterial^3^. No estudo de Wowerm et al.^28^, foram comparados esses e outros parâmetros da PPG com IA e VOP obtidos por tonometria de aplanação. O IA obtido da PPG foi a variável mais relacionada ao IA e à VOP obtidos por tonometria.

A VOP é avaliada pelo método de tonometria de aplanação, em que sensores de pressão são colocados em artérias superficiais para avaliar a rigidez arterial, tendo como exemplos as artérias carótida, radial e femoral, considerando-se como padrão-ouro a VOP carótida-femoral^28^. Apesar de a VOP ser o padrão-ouro para avaliação da rigidez arterial, o método de tonometria é mais incômodo e de difícil operação. Como alternativa, tem-se a PPG, que usa uma tecnologia mais barata e não operador dependente, tornando-a mais viável e adequada na prática clínica^28^^,^ 29.

A VOP é considerada um biomarcador de degeneração vascular. Acompanha o mesmo ciclo do batimento cardíaco, em que uma onda de pulso é gerada e percorre o leito arterial até encontrar resistência periférica. A resistência arterial dependerá do nível de complacência que o vaso oferece^29^. A VOP pode ser derivada de parâmetros obtidos da PPG, conforme demonstrado por Millasseau et al.^30^ Assim, em indivíduos jovens existe uma melhor complacência vascular, devido à maior elasticidade das artérias, e portanto a onda refletida é lenta, com um intervalo (ΔT) maior entre pressão sistólica e diastólica e com um entalhe dicrótico^29^. Já em pacientes mais idosos, devido ao envelhecimento vascular e a comorbidades como HAS e DM, ocorre um aumento na VOP, gerando um ΔT menor e um entalhe dicrótico pouco perceptível^29^. O IR é um parâmetro que normaliza o ΔT de acordo com a altura do indivíduo, possuindo a mesma grandeza (velocidade) e unidade (m/s) que a VOP^3^.

Assim, a VOP tem forte correlação com a idade e a PA, pois ambas diminuem a complacência vascular, gerando um aumento da rigidez arterial. Diretrizes atuais de HAS vêm recomendando a necessidade de usar biomarcadores como coadjuvantes na acurácia da estratificação de risco cardiovascular^29^.

Na comparação com a idade, foi observado que, quanto maior a idade, pior o resultado obtido em relação aos valores de referência do ITB e também em relação aos parâmetros obtidos na PPG. Esse resultado revela que houve um aumento do índice de rigidez arterial, demonstrando que esses indicadores podem ser utilizados como marcadores de envelhecimento arterial^2^.

Em relação à comparação entre ITB e variáveis da PPG, não foi encontrada associação significativa. Isso se deve ao perfil dos pacientes do estudo, em que a maioria pertence a um grupo de risco menos propenso a ter DAP, pois a mediana de idade foi baixa, considerando-se o grupo em estudo, e, conforme mencionado anteriormente, quanto maior a idade, menor seria o ITB. Da mesma forma, a percentagem de fumantes foi pequena, e sabe-se que ser tabagista também levaria a uma redução do ITB^2^. Com isso, foi verificado que a doença vascular tem relação direta com a idade, no sentido de que, quanto mais idade, maior tendência a ter aterosclerose, sendo isso o que foi mostrado com o ITB e os índices da PPG^2^.

Nesse trabalho, na comparação entre ITB e parâmetros da PPG, esperava-se que houvesse uma correlação forte entre eles, o que não foi possível, por se tratar de uma amostra saudável com indivíduos sem doença periférica grave e com ITB dentro da normalidade. Apesar de não ter havido correlação significativa, foi observada associação entre idade, ITB e parâmetros da PPG, revelando que o envelhecimento arterial foi também evidenciado na PPG, mostrando que são necessários mais estudos para que esses dados possam ser confirmados.

Similar ao ITB com EOA, a PPG com o uso de um oxímetro de pulso, por ser prática e de simples manuseio, pode se tornar um instrumento acessível na prática clínica, constituindo um método não invasivo na detecção precoce da doença cardiovascular^20^.

Na regressão linear multivariada, foi demonstrado que mulheres mais idosas e tabagistas são mais propensas a possuir um menor ITB. Esses achados são corroborados pela literatura, a qual aponta que fatores como envelhecimento e hábito tabágico estão fortemente associados à aterosclerose. E, ao contrário do que se acreditava, as mulheres apresentam taxas de DAP igual ou superior à dos homens, principalmente após a menopausa, pela redução hormonal^1^^,^ 31. Esperava-se que os parâmetros da PPG estivessem envolvidos, mas não foi encontrada associação com o ITB. Por outro lado, o estudo de Allen et al.^2^, também utilizando técnicas de extração de sinal da PPG, demonstrou boa acurácia na estratificação de pacientes com DAP^21^.

Dentre as limitações do estudo, podem ser descritas a não avaliação prévia de sintomas de DAP e a normalidade do ITB de grande parte da amostra, compreendida por idosos saudáveis, o que dificultou a comparação com os índices da PPG. Além disso, a utilização do EOA para a obtenção dos dados de PA pode ter sido uma fonte de viés, apesar da indicação e da simplicidade de seu uso^20^.

## CONCLUSÃO

Foi demonstrado que os valores do ITB estiveram em sua maioria dentro da normalidade. Apesar de ser encontrada uma correlação entre idade, ITB e parâmetros da PPG, indicando envelhecimento arterial, o ITB não esteve associado aos indicadores da PPG. O modelo preditivo demonstrou que mulheres, indivíduos com idade mais avançada e tabagistas apresentam menor ITB.

De qualquer forma, os resultados deste trabalho demonstram o potencial uso da PPG como técnica não invasiva. Entretanto, ainda são necessários mais estudos para a padronização da avaliação vascular por meio de curva da PPG e para sua utilização no diagnóstico e rastreamento de DAP.
